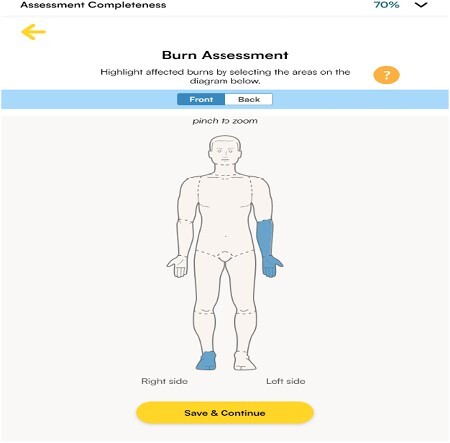# 606 Artificial Intelligence-powered Mobile Tool for Burn Injury Evaluation for First Responders

**DOI:** 10.1093/jbcr/irae036.240

**Published:** 2024-04-17

**Authors:** Alexander Perry, Shawn Dodd, Hannah O Chan, Rakesh Joshi, Joshua N Wong, Collin Hong

**Affiliations:** University of Alberta, Edmonton, AB; Skinopathy, Toronto, ON; University of Alberta Burn Centre, Edmonton, AB; University of Alberta, Edmonton, AB; Skinopathy, Toronto, ON; University of Alberta Burn Centre, Edmonton, AB; University of Alberta, Edmonton, AB; Skinopathy, Toronto, ON; University of Alberta Burn Centre, Edmonton, AB; University of Alberta, Edmonton, AB; Skinopathy, Toronto, ON; University of Alberta Burn Centre, Edmonton, AB; University of Alberta, Edmonton, AB; Skinopathy, Toronto, ON; University of Alberta Burn Centre, Edmonton, AB; University of Alberta, Edmonton, AB; Skinopathy, Toronto, ON; University of Alberta Burn Centre, Edmonton, AB

## Abstract

**Introduction:**

Accurate assessment and early interventions in the field are crucial to the prognosis of burn injuries. Studies have indicated that up to 35% of burn patients are inappropriately transferred to hospitals. In some pediatric burns, reports have suggested the total burn surface area (TBSA) has been overestimated as much as 44%. First responders play a pivotal role in the assessment and management of burn injuries, especially in remote areas. An intuitive mobile application that incorporates the standardized practices of Advanced Burn Life Support (ABLS) with integrated artificial intelligence (AI) to assist in burn size, depth, and management is needed.

**Methods:**

The mobile application was designed with an experienced team of burn specialists, physicians, and software engineers to identify the gaps in first responder burn care and to standardize methods for initial burn assessment. Previously assessed burn photos were characterized based on their depth into split partial thickness, deep partial thickness, and full thickness burns. Laser doppler imaging taken at the time of clinical assessment confirmed the burn depth. These images were used to build a convolutional neural network from to predict burn depth and boundaries.

**Results:**

An AI-integrated mobile application was developed encompassing a primary survey with management solutions with the fundamentals based on ABLS. The application ensures the collection of pertinent information for burns, such as the patient's weight for calculating fluid resuscitation and provides recommendations based on burn depth and surface area. Photos taken using the mobile device can be analyzed by the AI in real time to aid in burn assessment. The accuracy of the prototype AI model can in distinguish severity assessment with an F1 accuracy score of 78% with a receiver operating characteristic of 85%. Furthermore, the model has a 92% accuracy for determining the boundaries of the burn.

**Conclusions:**

A smartphone burn application provides an integrated way to improve the efficiency and accuracy of first responders to assess, manage, and triage burns before reaching the hospital. Through the application, management recommendations are tailored to the extent of the injury and provides a detailed report to secondary healthcare providers.

**Applicability of Research to Practice:**

A state-of-the-art burn application can be a tool that can be used by first responders to capture essential data while ensuring the accuracy of the initial assessment. The integration of artificial intelligence will help personalize and streamline the management pathway improving communication between field and hospital care providers and improving patient care.